# Molecular characterization of *Leishmania* species from stray dogs and human patients in Saudi Arabia

**DOI:** 10.1007/s00436-021-07166-z

**Published:** 2021-05-04

**Authors:** Abdullah D. Alanazi, Abdulazi S. Alouffi, Mohamed S. Alyousif, Abdulsadah A. Rahi, Magda A. Ali, Hend H. A. M. Abdullah, Fabio A. Brayner, Jairo Alfonso Mendoza-Roldan, Marcos Antonio Bezerra-Santos, Domenico Otranto

**Affiliations:** 1 Department of Biological Sciences, Faculty of Science and Humanities, Shaqra University, P.O. Box 1040, Ad-Dawadimi, 11911 Saudi Arabia; 2 King Abdulaziz City for Science and Technology, Riyadh, Saudi Arabia; 3 Department of Zoology, College of Science, King Saud University, Riyadh, Saudi Arabia; 4 Department of Biology, College of Science, University of Wasit, Kut, Wasit 00964 Iraq; 5 Department of Parasitology and Animal Diseases, Veterinary Research Division, National Research Centre, 33 Bohouth St., Dokki, Giza, 12622 Egypt; 6 Laboratório de Imunopatologia Keizo Asami, Universidade Federal de Pernambuco, CEP, Recife, 50670-901 Brazil; 7 Dipartimento di Medicina Veterinaria, Università degli Studi di Bari, Bari, Italy; 8 Faculty of Veterinary Sciences, Bu-Ali Sina University, Hamadan, Iran

**Keywords:** *Leishmania tropica*, *Leishmania major*, Dogs, Patients, kDNA, nPCR, Saudi Arabia

## Abstract

*Leishmania major* and *Leishmania tropica* cause cutaneous leishmaniasis in humans and dogs in several parts of the world, with a large number of cases recorded in the Middle East. However, when they occur in sympatry, the role of each species of *Leishmania* in the epidemiology of cutaneous leishmaniasis (CL) is not clear. To assess the frequency and to identify the species of *Leishmania* that infect humans and stray dogs in Riyadh and Al-Qaseem (Saudi Arabia), 311 stray dogs and 27 human patients who were suspected for *Leishmania* infection were examined for CL by a nested polymerase chain reaction (nPCR). Seven (25.9%) out of 27 human patients scored positive for *Leishmania* spp. (i.e., *L. major* in five patients from Riyadh and *L. tropica* in two patients from Al-Qaseem). Out of 311 dogs, five (1.6%) were infected by *L. tropica*. Data herein presented demonstrate the occurrence of *L. tropica* in dogs and humans in Saudi Arabia, as well as the occurrence of *L*. *major* in humans.

## Introduction

Leishmaniases are a complex group of diseases caused by protozoa of the genus *Leishmania*, which are included in the group of neglected tropical diseases affecting mainly vulnerable human populations worldwide (WHO 2018). *Leishmania* spp. are transmitted by phlebotomine sand flies of the genus *Phlebotomus* in the Old World and *Lutzomyia* in the New World (Dantas-Torres et al. 2012). The disease caused by these protozoa is classified as cutaneous (CL), visceral (VL), and mucocutaneous (MCL) leishmaniases, all of which have been reported in Saudi Arabia (Abuzaid et al. 2017; Sirdar et al. 2018; Hawash et al. 2018). Moreover, CL caused by *Leishmania major* has been reported in that country, with the highest prevalence being recorded in the Riyadh, Qassim, Al-Madinah, Al-Hassa, Hail, and Asir regions (Al-Tawfiq and AbuKhamsin 2004; Amin et al. 2013; Alanazi et al. 2016), where more than 26,300 cases had been estimated from 2006 to 2016 (Abuzaid et al. 2017). In addition, there are several reports of leishmaniasis caused by *Leishmania infantum*, *L. major*, and *Leishmania tropica* in humans and wild animals in Saudi Arabia (Peters et al. 1986; Elbihari et al. 1987; Al-Zahrani et al. 1988a, 1988b; Alanazi et al. 2019a, 2019b).

In western Saudi Arabia (Al-Madinah Al-Munawarah province), CL was diagnosed in human patients by internal transcribed spacer 1 (ITS-1), polymerase chain reaction (PCR), and restriction fragment length polymorphism (RFLP) (El-Beshbishy et al. 2013a, 2013b). The PCR screening established *L. major* and *L. tropica* as the causative agents for the above infection, with a kDNA PCR sensitivity of 90.7% and of ITS-1 PCR of 70.1%. Additionally, *Leishmania* spp. were detected in human patients in Al-Qaseem province, central Saudi Arabia, with prevalence of 49.5% for *L. major*, 28.6% for *L. tropica*, and 3.9% for *L. infantum* (Rasheed et al. 2019).

Despite the availability of several molecular studies that report the diagnosis and identification of *Leishmania* species worldwide (Ferreira et al. 2008; Akhavan et al. 2010; Toz et al. 2013; Silva et al. 2017), in Saudi Arabia, information on CL in human patients and on dog populations from endemic areas is still scant. Therefore, the aim of this study is to detect and identify the *Leishmania* spp. infecting humans and stray dogs in Al-Qaseem province and in Riyadh city, Saudi Arabia, providing data for a better understanding of the epidemiology of the infection in the study area.

## Material and methods

### Study areas, sampling, and DNA isolation

**Fig. 1:**
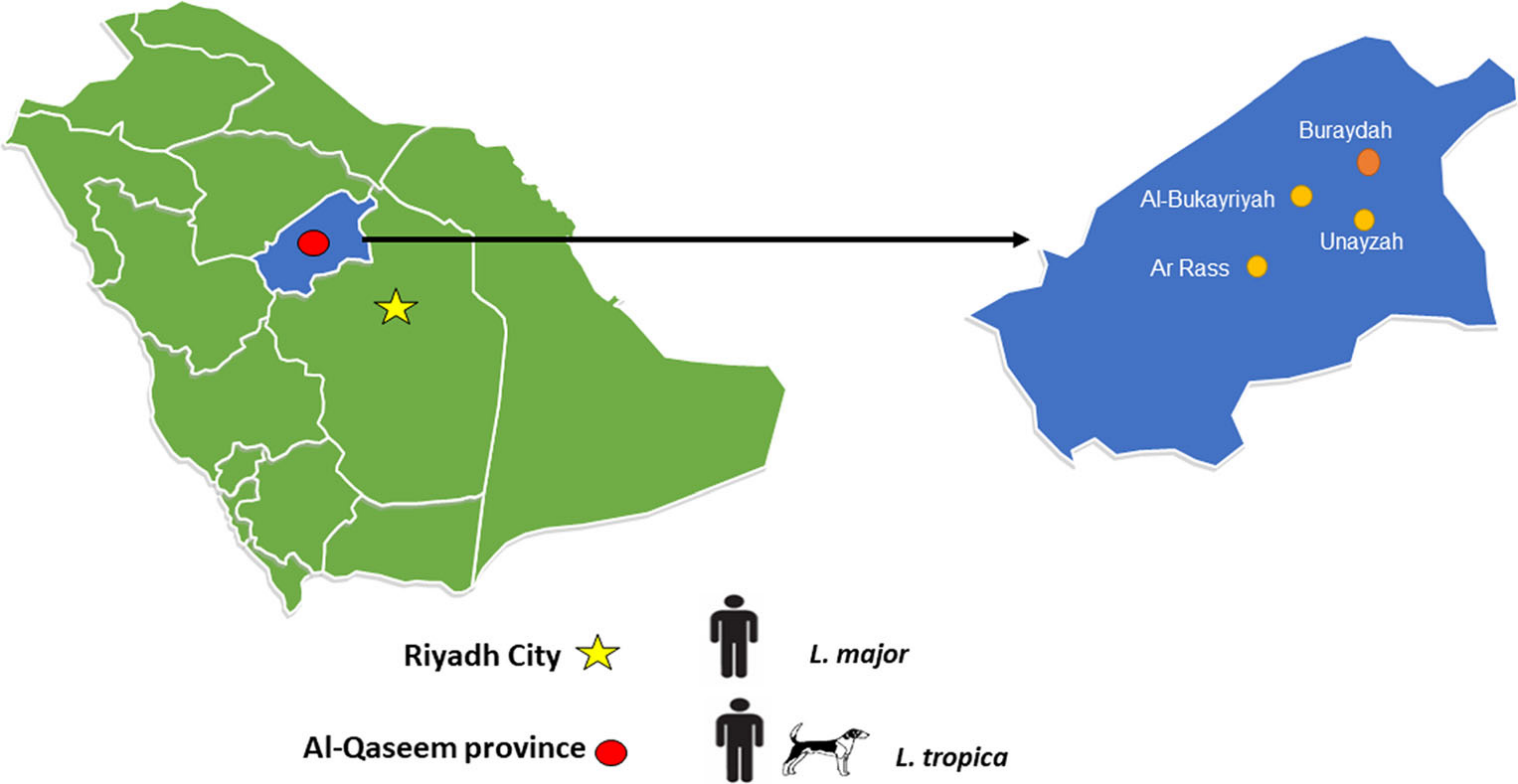
Map showing the location of the study areas in Saudi Arabia

The investigation was conducted from January 2018 to May 2019 in Al-Qaseem province (latitude 25–23° N and longitude 42–24° E) and Riyadh city (latitude 24–08°N and longitude 47–18° E), Saudi Arabia (Fig. 1). A total of 27 human patients who were suspected to be infected by *Leishmania* species were seen in either the King Saud Medical City in Riyadh city (*n* =16) or Buraydah Central Hospital (*n* =11) in Al-Qaseem province. The presence of *Leishmania* was investigated in all samples, which were collected after clinical examination (Akilov et al. 2007). Briefly, skin biopsies (i.e., 5–10 mm in diameter) were taken under sterile conditions from the border of the ulcerous and cutaneous lesions, and DNA samples were extracted from all biopsies by MagNaA pure DNA extraction through the use of a Pure LC DNA Isolation Kit (Roche Applied Science, Germany) according to the manufacturer’s instructions.

From January 2018 to May 2019, 311 stray dogs were trapped in Al-Qaseem province by bait traps (Havahart®) and were examined physically for canine leishmaniasis skin lesions. Seven dogs were suspected of infection with canine leishmaniasis due to the presence of cutaneous nodules or ulcerated lesions . Skin biopsies (5mm in diameter) were collected under sterile conditions from the borders of the ulcers and were inoculated into M199 medium (Gibco, Life Technologies, Germany), which was supplemented with 25 mmol/l of 4-(2-hydroxyethyl)-1-piperazineethanesulphonic acid (HEPES) (pH:7.5) and 20% fetal bovine serum (Gibco, Life Technologies, Germany). These samples were then incubated at 24 °C. Ten days after sample incubation, parasites were harvested and washed with ice-cold phosphate-buffered saline (10X PBS, pH: 7.4) and stored at −20°C before DNA isolation. DNA from parasite culture was isolated by use of the ReliaPrep™ gDNA Tissue Miniprep System Kit (Promega, Madison, USA), following the manufacturer’s instructions.

### *Leishmania*-nested PCR

DNA samples from humans and dogs were screened via nested PCR. Initial amplification was performed with the primers (CSB2XF: 5′-ATTTTTCGCGATTTTCGCAGAAACG-3′) and (CSB1XR: 5′-CGAGTAGCAGAAACTCCCGTTCA-3′). The set of primers (13Z: 5′-ACTGGGGGTTGGTGTAAAATAG-3′) and (LiR; 5′-TCGCAGAACGCCCCT-3′) were applied for the second step (Noyes et al. 1998)*.* These primers amplify kDNA fragments of ~680bp for *L. infantum*, ~750bp for *L. tropica*, and ~560bp for *L. major* (Noyes et al. 1998)*.*

The preparation of the PCR master mix was performed using the AccuPower® PCR PreMix kit (Bioneer, Daejeon, Korea). The prepared PCR pre-mix volumes containing potassium chloride (KCl) at a concentration of 30mM, magnesium chloride (MgCl2) at 1.5mM, tris(hydroxymethyl)aminomethane hydrochloride (Tris-HCL at pH 9.0) at 10mM, Taq DNA polymerase, and deoxynucleoside triphosphate (dNTP) were adjusted to 2μl. In addition, 1μl of each initial CSB2XF and CSB1XR primers at concentrations of 10pmol/ul (Bioneer, Daejeon, Korea) and 3μl of DNA were added to the reaction mixture. Finally, 13μl of deionized water (ddH2O) were added up to a total volume of 20μl for reaction. Negative control was included in the final nPCR. The reaction was performed in a thermal cycler (Techne TC-3000, USA) according to the following conditions: initial denaturation temperature of 94°C for 5min, 30 cycles of denaturation at 94°C for 30s, annealing at 55°C for 60s, extension at 72°C for 60s, and final extension at 72°C for 7min; and then the reaction was held at 4°C.

The second step of PCR involved 13Z and LiR primers, and the same PCR master mix except that 3μl of template PCR product from the first reaction was used. In this second round, PCR products that were obtained were electrophoresed on a 1.5% agarose gel containing 1μl Syber safe (Thermo Scientific™, Nalgene, UK) in tris-acetate–ethylenediaminetetraacetic acid (EDTA) buffer (50X) at 100V for 45min and visualized under a UV imaging system (ImageQuant Laz4000, GE Healthcare Life Science, Hammersmith, UK).

### *Leishmania* kDNA sequencing and BLAST analysis

The amplified products of *Leishmania* species were sequenced, and the results were compared with the sequences available in the GenBank database via BLAST search tool (http://blast.ncbi.nlm. nih.gov/). The obtained sequences were aligned with a set of reference sequences that were available in GenBank using CLUSTALW in MEGA software version 7.0 (Kumar et al. 2016). The phylogenetic tree was constructed using the maximum-likelihood method and with the Hasegawa-Kishino-Yano (HKY) model with 2000 bootstrap replicates in MEGA 7.0 software, using *Trypanosoma cruzi* (AJ748063) as outgroup (Kumar et al. 2016; Al-Bajalan et al. 2018).

## Results

Of the 27 human patients who were examined, five out of 16 (31.2%) from Riyadh and two out of 11 (18.2%) from Al-Qaseem were positive for *Leishmania* spp. Sequence analysis of the *Leishmania* kDNA confirmed that the five positive human samples from Riyadh were all *L. major* with nucleotide identity ranging from 99.3 to 100% with *L. major* sequences from Iraq (MN313423). The *Leishmania* sequences from the two-positive human samples from Al-Qaseem presented identity of 99.7 to 100% with *L. tropica* from Iraq (MF166799).

Of 311 dogs, seven (2.3%) presented cutaneous lesions (i.e., 1.5 × 5 cm) in different anatomical sites (e.g., nose, muzzle, abdomen, and interdigital spaces), and five of them were positive for *L*. *tropica* (Table 1), presenting nucleotide identity ranging from 99.33 to 99.80% with sequences of *L. tropica* from Iraq (MF166800, MN334661) and the UK (AF308689).

**Table 1:** Data of dogs that were suspected to carry canine leishmaniasis and that were trapped in Al-Qaseem province

Dog ID	Site of trapped dogs	Sex	Age (years)	Location of cutaneous lesions	*Leishmania tropica*
Dog No.1	Unayzah	Male	2	Left ear	Neg
Dog No.2	Al Bukayriyah	Male	1	Nose	Pos
Dog No.3	Buraydah	Male	4	Abdomen	Neg
Dog No.4	Al Bukayriyah	Female	2	Under mouth lips	Pos
Dog No.5	Buraydah	Male	3	Muzzle	Pos
Dog No.6	Ar Rass	Female	3	Nose	Pos
Dog No.7	Buraydah	Male	2	Upper right leg	Pos

**Fig. 2:**
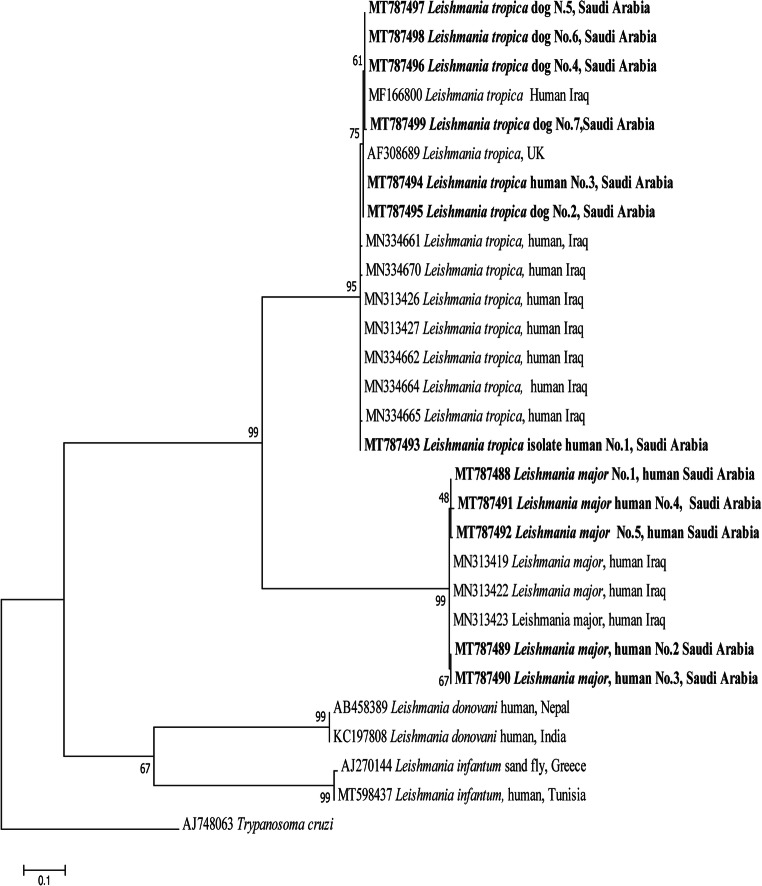
Maximum likelihood Kinetoplast DNA-based phylogenetic analysis of genotypes identified in this study. Phylogenetic tree highlights the position of *Leishmania* spp. of the present study (bold) using 2000 bootstrap replicates and *Trypanosoma cruzi* as outgroup

The phylogenetic tree for *L. major* and *L. tropica* sequences from human samples clustered with sequences from Iraq, and those for *L. tropica* from dog samples clustered with sequences from Iraq and the UK (Fig. 2). In addition, the isolates of *L. tropica* that were taken from humans and dogs in the present study were closely related (i.e., 98.60 to 99.65% with query cover that ranged from 98.20 to 99.50%) to kDNA of *L. tropica* (Saudi strain, MHOM/SA/91/WR1063) that were recorded on GenBank under the accession number X84845.1. Representative sequences of *L. major* and *L. tropica* that were retrieved in the present study were deposited in the GenBank database under the accession numbers MT787488 to MT787499.

## Discussion

This study provides molecular evidence of the circulation of *L. major* and *L. tropica* in human and dog populations from the investigated areas. The above *Leishmania* spp. have already been recorded as agents of cutaneous leishmaniasis in Saudi Arabia and Middle Eastern countries (Bamorovat et al. 2015; Al-Salem et al. 2016; Baneth et al. 2016; Al-Bajalan et al. 2018). However, here we provide data on the occurrence of *L. tropica* in humans and dogs from the central part of Saudi Arabia. This infection has been previously reported in the west and south west of Saudi Arabia in association with the distribution of *Phlebotomus sergenti*, a proper vector for *L. tropica* (Al-Zahrani et al. 1988a, 1988b). Conversely, *L. major* is more prevalent throughout the country and can be found in the open desert regions of Saudi Arabia (Abuzaid et al. 2017; Haouas et al. 2017). Previous studies performed in Saudi Arabia have reported the natural infection by *L. major* in dogs through the use of enzymatic biochemical methods (Elbihari et al. 1984; Peters et al. 1985), though in these studies, no clinical information was available nor were serology or molecular confirmation performed.

The high similarities of the nucleotides of human *L. major* and *L. tropica* isolates with those of Iraq (accession numbers MN313423 and MF166799) and of the dog *L. tropica* isolates with those of Iraq and UK (accession numbers MN334665, MF166799, and AF308689, respectively) were confirmed by phylogenetic analysis. Moreover, this study showed that *L. tropica* from humans and dogs was closely related with the kDNA of *L. tropica* samples from the Middle East. This might be due to the distribution of similar sand fly species in the different parts of Saudi Arabia and the Middle East, which may act as proper vectors of both *Leishmania* spp. (Al-Salem et al. 2016; Du et al. 2016). In the phylogenetic tree, *L. tropica* and *L. major* clustered in separate clades, distinct from the *L. donovani* complex (i.e., *L. infantum* and *L. donovani*). Moreover, *L. tropica* sequences presented very limited intra-specific genetic diversity, unlike the sequences that were previously classified as belonging to the *L. donovani* complex (Medkour et al. 2020).

Though CL is endemic in many parts of Saudi Arabia, the paucity of data concerning the relationship between the disease, the vectors, and the reservoirs is a major hindrance to the comprehension of the transmission cycles, particularly given that the distribution patterns can easily change through the years in specific geographical areas (Mendoza-Roldan et al. 2020). Data herein reported contribute to the filling of existing gaps in knowledge to increase the awareness of the Ministry of Health in Saudi Arabia to prevent outbreaks and the spread of CL.

## Conclusion

This study confirms the occurrence of *L. major* and *L. tropica* in humans and *L. tropica* in dogs from Al-Qaseem province and Riyadh City, Saudi Arabia. However, the relationship between sand fly vectors and reservoirs of this disease remains unclear, advocating further studies in these areas.

## Declarations

This study was reviewed and approved by the Ethics Committee of the Department of Biological Sciences at the Shaqra University, according to the ethical principles of animal research (protocol SH 2-2017).
